# Small RNA sequencing revealed various microRNAs involved in ethylene-triggered flowering process in *Aechmea fasciata*

**DOI:** 10.1038/s41598-020-63597-1

**Published:** 2020-04-30

**Authors:** Yuanhao Ding, Jiabin Wang, Ming Lei, Zhiying Li, Yonglin Jing, Haiyan Hu, Sitao Zhu, Li Xu

**Affiliations:** 1 Institute of Tropical Crop Genetic Resources, Chinese Academy of Tropical Agricultural Sciences, Danzhou, 571737 Hainan China; 2 Ministry of Agriculture Key Laboratory of Crop Gene Resources and Germplasm Enhancement in Southern China, Chinese Academy of Tropical Agricultural Sciences, Danzhou, China; 3 Hainan Key Laboratory for Sustainable Utilization of Tropical Bioresources, Institute of Tropical Agriculture and Forestry, Hainan University, HaiKou, 570228 China; 4 BGI-WuHan, BGI-ShenZhen, WuHan, 430074 China

**Keywords:** miRNAs, Flowering

## Abstract

Ethylene-triggered flowering is a common phenomenon in plants of the family Bromeliaceae, but its molecular mechanism remains unclear. As a classical group of small RNAs, microRNAs play an essential role in the regulation of flowering. In this study, we found that various miRNAs participate in the ethylene-triggered flowering process in *Aechmea fasciata* via small RNA sequencing using juvenile and adult plants treated with ethylene for 24 hours. Finally, 63 known miRNAs, 52 novel miRNAs and 1721 target genes were identified or predicted. Expression changes of specific miRNAs were validated by qRT-PCR and northern blotting. Some predicted targets, including *SPL*, *GAMYB* and *ARF*, were verified in RLM-RACE experiments. Gene Ontology (GO) and KEGG analysis showed that numerous developmental and RNA-related processes were enriched. Integrated analysis of the transcriptomic data with small RNA sequencing revealed that numerous miRNAs and targets involved in ethylene-triggered flowering in *A. fasciata*. Our study is helpful for illuminating the molecular basis of the ethylene-triggered flowering phenomenon in Bromeliaceae.

## Introduction

*Aechmea fasciata*, a typical ornamental plant of the family Bromeliaceae, is widespread throughout the tropics and subtropics. The flower of *A*. *fasciata* is the most valuable ornamental part of the plant. Flowering of *A*. *fasciata*, similar to that of other species in the *bromeliad* family such as pineapple (*Ananas comosus*), can be induced by the gaseous plant hormone ethylene^1^. Although ethylene and its alternatives have long been used to control the flowering time of bromeliads, the molecular mechanism of the effect remains unclear. Thus, dissection of the molecular basis of the ethylene-triggered flowering of *A*. *fasciata* will be helpful in artificially controlling flowering time during large-scale industrial production. In addition, *A*. *fasciata* may also share the similar crassulacean acid metabolism (CAM) pathway with other bromeliads. Because its life cycle is much shorter than that of pineapple and most other bromeliads, *A*. *fasciata* is a relatively convenient model plant for ethylene-triggered flowering process or CAM pathway researches of bromeliad.

For natural out-of-season flowering, ethephon (2-chloroethylphosphonic acid) is the chemical that is most widely used to induce synchronized flowering in the production of most bromeliads. In early research, ethylene was considered the most important phytohormone in inducing flowering in pineapple. Foliar application of aminoethoxyvinylglycine ([S]-trans-2-amino-4-(2-aminoethoxy)-3-butenoic acid hydrochloride, AVG), which inhibits ACC synthase and thereby reduces ethylene biosynthesis^2^, can prevent the natural flowering of pineapple^3,4^. Silencing of *ACACS2*, an ACC synthase gene involved in ethylene biosynthesis, also caused markedly delayed flowering of pineapple under natural conditions^5^. Recently, transcriptomic analyses of pineapple have shown that ethylene response genes and flowering-related genes such as *Ethylene Response Factor* (*ERF*), *Ethylene-Resistant* (*ETR*), *Ethylene-Insensitive* (*EIN*), *Flower Locus T* (*FT*), *PISTILLATA* (*PI*), *VERNALIZATION* (*VRN*) and *APETALA 2* (*AP2*) are differentially expressed after ethylene treatment^6,7^. Furthermore, the *AcFT*, *AcPI* and *AfAP2* genes of pineapple and *A*. *fasciata* have been reported to affect the flowering process^8–10^. In addition, it was reported that obvious floral bud differentiation occurs within 72 hours after Ethrel^®^ 48 treatment, but changes in the levels of expression of the *AcACS2*, *AcFT*, and *AcLFY* genes could be detected in the first 24 hours^11^.

In *Arabidopsis*, environmentally activated ethylene signals can reduce bioactive gibberellin acid (GA) levels by inhibiting CONSTITUTIVE TRIPLE RESPONSE1 (CTR1) and increasing Ethylene-Insensitive 3 (EIN3)^12,13^. In turn, DELLA accumulation is enhanced, resulting in repression of the flora meristem identity genes *LEAFY* (*LFY*) and *SUPPRESSOR OF OVEREXPRESSION OF CONSTANS 1* (*SOC1*)^13^. The loss-of-function mutation (*ctr1-1*) of *AtCTR1*, a key negative regulator kinase gene in the ethylene response, also caused a delayed flowering phenotype^13^. However, bolting time was delayed in various ethylene-related mutants such as *ein3-1*, *ein2-1* and *etr1*, indicating that ethylene accelerates flowering in *Arabidopsis*^14^. In rice, overexpression of *ETHYLENE RESPONSE 2* (*ETR2*) (the subfamily II ethylene receptor gene) delayed the floral transition, and RNA interference (RNAi) or *Osetr2* mutant plants exhibited an early-flowering phenotype^15^. In addition, the *Osctr2* rice mutant and *35S:OsCTR2*^1–5^,^13^] transgenic lines both showed delayed heading^16^. These findings imply that the activation of ethylene signalling may enhance the floral transition of rice. Considering the positive effect of ethylene on flowering in bromeliads, ethylene may function in different pathways to regulate the floral transition in different species.

MicroRNAs (miRNAs), as a classical group of small RNAs, play an essential role in the post-transcriptional regulation of gene expression and are involved in diverse developmental processes and stress responses. Many miRNAs have been identified in various Bromeliaceae, including pineapple and *Vriesea carinata*^17–19^. Recently, various miRNAs were found to be involved in CAM and in photosynthesis pathways in pineapple^20^. However, there are no reports on a possible role of miRNAs in ethylene-triggered flowering processes in bromeliads. In *Arabidopsis*, it was discovered earlier that miR172, miR156, and miR159 are involved in the flowering process by regulating the expression of their targets *AP2*, *SQUAMOSA PROMOTER-BINDING PROTEIN-LIKE* (*SPL*), and *MYB3*^21–23^. In banana, a miRNAome of the banana fruit in response to ethylene or 1-MCP revealed that 82 differentially expressed miRNAs were found to be closely associated with the ripening process^24^. An *APETALA2* (*AP2*)-like gene from *A*. *fasciata*, *AfAP2-1*, was also shown to delay the flowering time of *Arabidopsis*^8^. Transcriptomic analysis of the ethylene-triggered flowering process in *A. fasciata* revealed that *AfAP2-1* was differentially expressed in adult plants after ethylene treatment^1^. Thus, we speculated that miRNA might participate in the flowering of *A. fasciata* under natural conditions and/or after ethylene treatment.

To identify the miRNAs involved in the flowering process in *A. fasciata*, adult and juvenile *A. fasciata* plants were treated at the same time with ethylene or with water. A total of 4 mixed samples from core leaves and shoot tips were harvested and used for small RNA sequencing, and 63 known miRNAs and 52 novel miRNAs were identified. Furthermore, 1721 target genes were predicted and validated in RLM-RACE experiments. Gene Ontology (GO) and KEGG pathway analysis was used to classify the diverse functions of the targets. Integrated analysis of the transcriptomic data with the small RNA sequencing data reveals that numerous miRNAs, including miR172, miR319 and miR529, appear to function in the ethylene-triggered flowering process in *A. fasciata*. We hypothesize that various miRNAs are involved in the ethylene-triggered flowering process in *A. fasciata*.

## Results

### Small RNA sequencing of *A. fasciata* after ethephon treatment for 24 hours

In *A. fasciata*, flowering is an age-dependent process. Juvenile plants cannot flower even if treated with ethephon, but adult plants can, and flowering can be induced by the phytohormone ethylene^1^. Thus, to reveal the roles of microRNAs in the ethylene-triggered flowering process, juvenile and adult plants of *A*. *fasciata* were treated with ethephon at 10:00 o’clock, and mock controls were synchronously treated with water. Core leaves and shoot apical meristems from 5 individual *A. fasciata* plants from each treatment were collected as samples for later small RNA sequencing. Total RNA was prepared as described in the Methods section and sent to Novogene (Beijing, China) for small RNA library construction and sequencing on an Illumina HiSeq. 2000 system.

**Figure 1:**
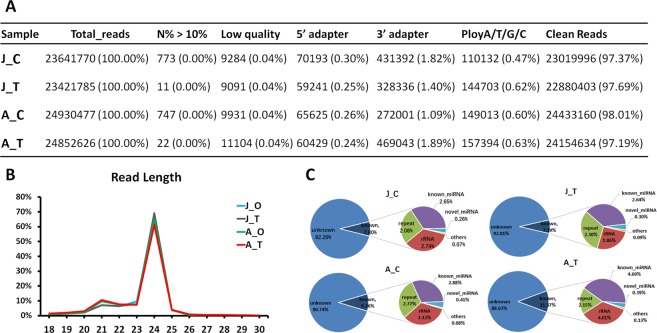
Reads analysis of small RNA sequencing data. (**A**) Quantity of raw and clean reads. Clean reads were generated from raw data by removed unqualified data. “J” indicates juvenile plants, “A” indicates adult plants, “C” indicates mock control (water treatment), and “T” indicates ethephon treatment; these designations are used similarly hereinafter. (**B**) Length distribution of 18–30 nt sRNAs in all samples. The highest-abundance sRNAs are 24 nt in length; the second highest-abundance sRNAs are 21 nt in length. (**C**) Annotation distribution of sRNAs. Nearly 90% reads were unknown sequences in all samples and known and novel miRNAs occupied nearly 3–4%.

After removing unqualified reads such as reads containing ‘N’, adapters, low- quality reads and poly A/T/G/C, 23 to 24 million clean reads were generated from each sample (Fig. 1A). Subsequently, the length of the small RNAs (sRNA) in each sample was determined. The distribution of RNAs 18–30 nt in length is shown in Fig. 1B. In all samples, 24-nt sRNAs show the highest abundance, followed by 21-nt sRNAs, consistent with the results of most previous sRNA sequencing studies^25,26^. The reads were then aligned to the GenBank and Rfam databases to annotate the rRNAs, tRNAs, trans-acting siRNAs (ta-siRNA), small cytoplasmic RNAs (scRNA), small nuclear RNAs (snRNA) and small nucleolar RNAs (snoRNA). After removing these sRNAs, microRNAs (miRNAs) were identified among the remaining sRNAs according to the methods described in the ‘Methods’ section. The final remaining unannotated sRNAs were labelled as ‘other’ sRNAs. The distribution of sRNAs is shown in Fig. 1C. Due to the lack of availability of a genome sequence, the majority of sRNAs (nearly 90%) were unannotated; rRNA and repeats made up nearly 5% of the total, 3–4% of the sRNAs were miRNAs, and the remaining sRNAs were tRNA, snRNA, etc.

### Identification of known and novel miRNAs in *A. fasciata*

**Figure 2:**
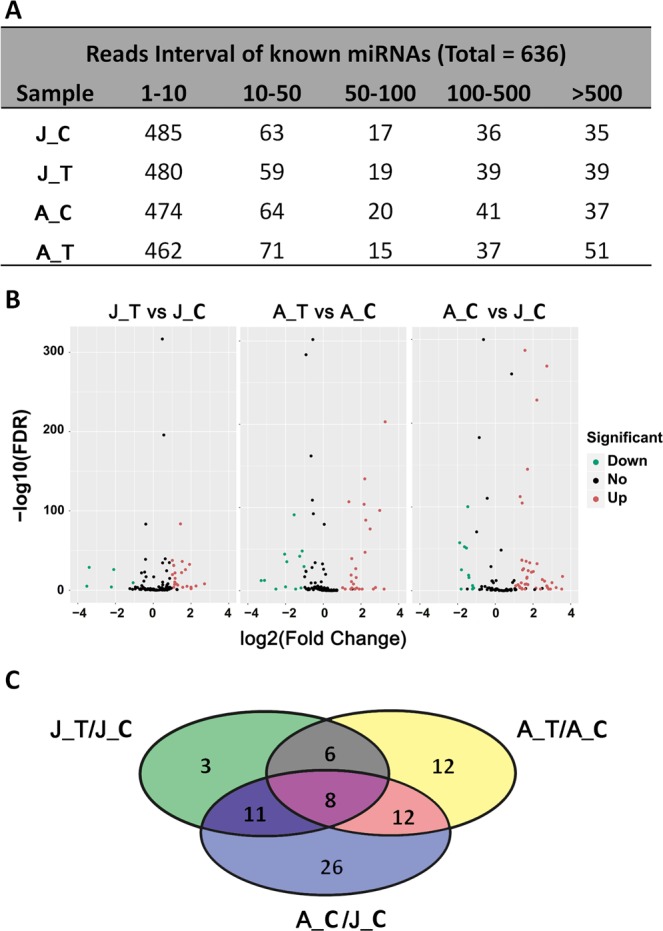
Identification of differentially expressed miRNAs related to the ethylene-triggered flowering process in *A. fasciata*. (**A**) Read intervals of known miRNAs obtained from miRBase. ‘1–10’, ’10–50’, etc. represent the read abundances of the miRNAs. (**B**) Volcano plot of differentially expressed miRNAs (DEMs). The screening conditions were FDR < 0.001 and |log_2_ (fold change)| > 1. Green, black and red dots represent down-, non- and up-regulated DEMs. (**C**) Distribution of DEMs in various combinations of samples. Eight DEMs were common detected in J_T/J_C, A_T/A_C and A_C/J_C.

Because no genome sequence is available for *A. fasciata*, we aligned the reads to the miRBase to identify known miRNAs. Subsequently, transcriptomic sequencing data from *A. fasciata* were used to predict the novel miRNAs^1^. Finally, a total of 636 known miRNAs were obtained from miRBase (Table S1). Although most of these miRNAs were expressed at low levels (less than 10 reads) (Fig. 2A), a considerable number of significant expressed miRNAs with more than 500 reads were identified according to previous studies^27^ (Fig. 2A). A total of 71 novel miRNAs were predicted according to the methods described in the section on bioinformatics analysis (Table S2). To ensure high quality of the data, we screened all miRNAs using the condition TPM > 1 in at least one sample. After this screening, 63 known miRNAs and 52 novel miRNAs remained (Tables S3, S4). The homologous miRNAs were then searched by blasting the mature nucleotide sequences to miRBase (Table S4). Most of the known miRNAs were 21 nt in length, but 24-nt miRNAs made up the highest fraction of the novel miRNAs (Tables S3, S4). Overall, a total of 27 miRNA families were found. The expression level of each miRNA family was calculated by summing the number of reads of each miRNA family member and is shown in Table S5. The results showed that miR159, miR166, miR319 and miR396 were the most enriched miRNA families, together representing nearly 80–90% of the reads (Table S5).

### Screening for differentially expressed miRNAs (DEMs) related to the ethylene-triggered flowering process in *A. fasciata*

To identify DEMs between samples, the changes in the expression of 115 filtered miRNAs (63 known miRNAs and 52 novel miRNAs) were calculated. The obviously changed DEMs were then screened using the conditions FDR < 0.001 and |log_2_ (fold change)| > 1 (Fig. 2B), yielding a total of 78 DEMs (Table S6). Most of these miRNAs (57/78) were differentially expressed in A_C (adult plants, control treatment) and J_C (juvenile plants, control treatment) (Fig. 2C). We speculated that these miRNAs might be related to the process of development of *A. fasciata* from juvenile to adult plants. Nearly half of them (38/78) were differentially expressed in A_T (adult plants, ethephon treatment) and A_C; relatively fewer miRNAs (28/78) were differentially expressed in J_T (juvenile plants, ethephon treatment) and J_C (Fig. 2C), indicating that more miRNAs are involved in the ethylene response in adult than in juvenile plants. In addition, there were 37 common DEMs in two or three comparing groups; this result implies that these miRNAs may have common regulatory roles in developmental or flowering processes.

### Expression analysis and validation of miRNAs

**Figure 3:**
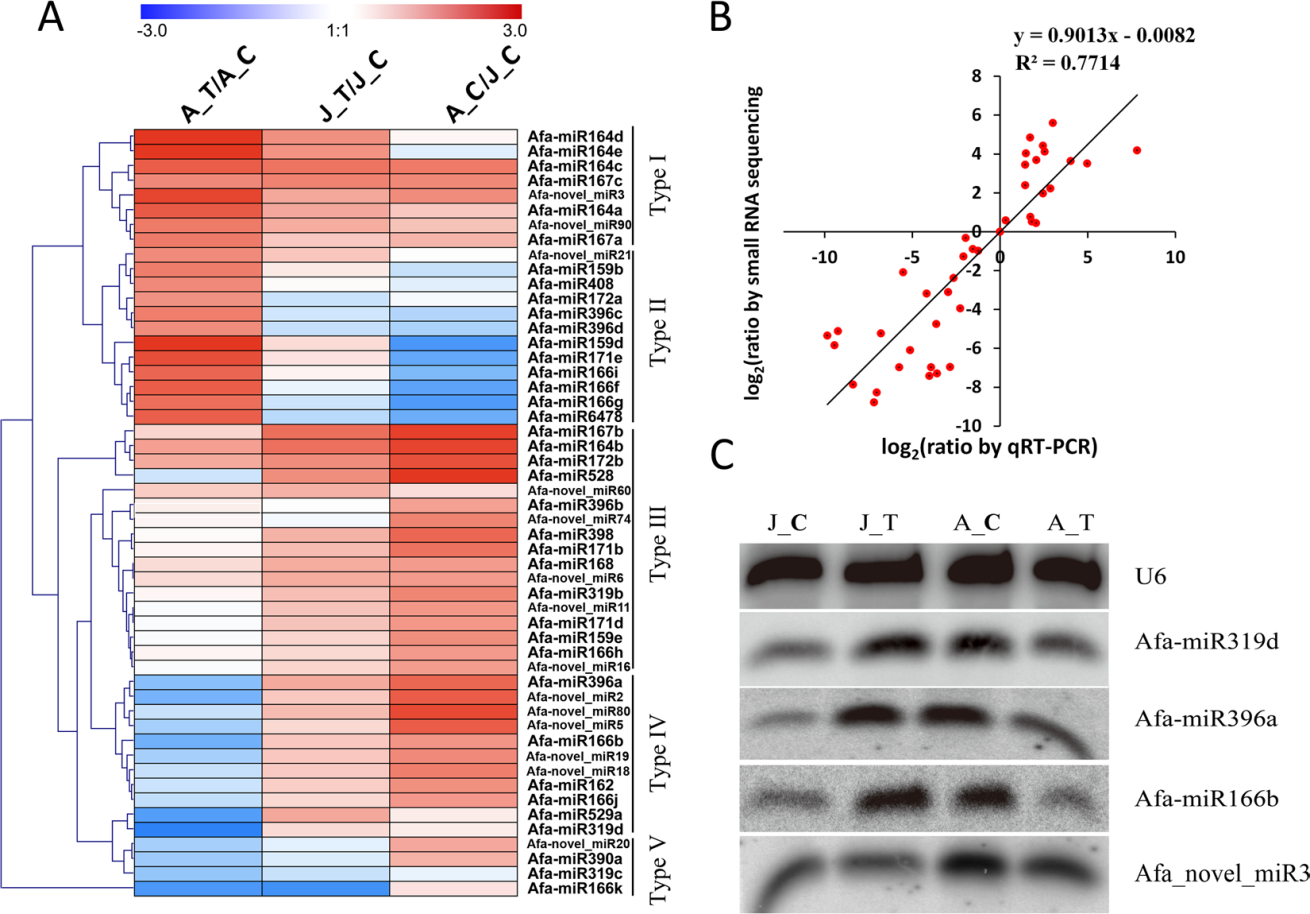
Heatmap analysis, qRT-PCR and northern blotting validation of miRNAs. (**A**) Heatmap analysis of DEMs showing the expression patterns of different samples. Five types of expression patterns were defined according to the expression patterns found in the samples. Type I: highly expressed both in A_T/A_C and J_T/J_C; Type II: highly expressed in A_T/A_C, reduced expression in A_C/J_C and low or reduced expression in J_T/J_C; Type III: low expression in A_T/A_C and J_T/J_C; Type IV: highly expressed in both A_T/A_C and J_T/J_C; Type V: reduced expression in both A_T/A_C and J_T/J_C. Red, white and blue blocks means miRNA up-, non- and down-regulated. (**B**) Correlation of expression profiles between small RNA sequencing and qRT-PCR. A total of 12 randomly selected miRNAs were analysed. (**C**) Northern blotting validation of miRNAs with relatively high expression levels. Three known miRNAs and 1 novel miRNA were chosen for northern blotting identification. The *U6* gene was used as an internal control.

To improve the accuracy of the data, miRNAs with low expression were removed, leaving 52 DEMs (38 known miRNAs and 14 novel miRNAs) with TPM > 10 (Table S7). A heatmap was prepared to show the specific expression patterns of these miRNAs in different samples (Fig. 3A). Finally, the miRNAs were classified into five different expression patterns (Type I to Type V) based on the expression patterns observed in the samples. The results showed that miRNAs of Type I, Type III and Type V had similar expression patterns in A_T/A_C and J_T/J_C, suggesting that these miRNAs may have similar functions in ethylene-triggered flowering in *A. fasciata*. Because miRNAs belonging to Type II and Type IV showed different expression patterns in A_T/A_C and J_T/J_C, we speculate that these miRNAs might function differently during the flowering process in *A. fasciata*.

To verify the reliability of the sequencing data, 12 miRNAs (8 known miRNAs and 4 novel miRNAs) were chosen at random and subjected to qRT-PCR. The measurements obtained by qRT-PCR were generally in agreement with the sequencing data (R^2^ = 0.7714, Fig. 2B). The relative expression of the miRNAs that were validated by qRT-PCR is shown in Fig. S1. In addition, some highly and differentially expressed miRNAs, including miR319, miR396, miR166 and the novel miRNA novel_miR3, were chosen for northern blotting validation (Fig. 3C). The results showed that miR396 and miR166 were both up-regulated in juvenile plants after ethylene treatment but displayed a completely opposite expression trend in adult plants (Figs. 3C, S1). Although miRNA novel_miR3 was highly up-regulated in the small RNA sequencing data (Fig. 3A), no difference was detected by northern blotting (Figs. 3C, S1). This difference might be due to inaccuracies in sequencing.

### Target prediction and RLM-RACE validation

**Figure 4:**
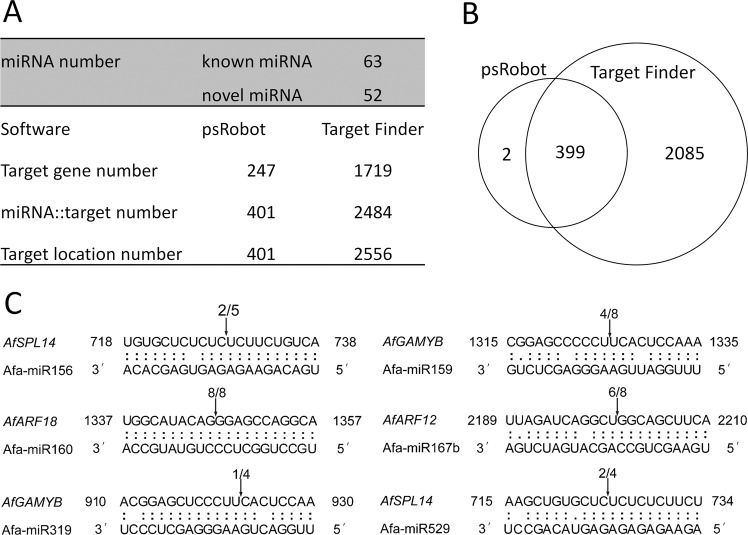
Target prediction of miRNAs and RLM-RACE validation. (**A**) Statistics of miRNAs and target genes predicted by psRobot and Target Finder. (**B**) Cleavage events distribution predicted by psRobot and Target Finder. (**C**) RLM-RACE identification of 6 chosen cleavage events. Arrows point out the cleavage sites. Numbers represent the clones examined and how many were corrected.

To further understand the functions of the miRNAs, their target genes were predicted using psRobot^28^ and Target Finder^29^. A total of 115 miRNAs (63 known miRNAs and 52 novel miRNAs, TPM > 1) were used to predict the target genes from the reported transcriptomic sequencing data of *A. fasciata*^1^. Finally, 247 and 1719 target genes, including 401 and 2484 cleavage events, were predicted through psRobot and Target, respectively (Fig. 4A). Among these, 399 common cleavage events were predicted by both the psRobot method and the Target Finder method (Fig. 4B). We then assembled all the cleavage events and target genes together in one set and finally obtained 2486 cleavage events and 1721 target genes (Table S8).

To verify the predicted targets, 6 cleavage events were chosen for RLM-RACE identification (Fig. 4C). From the results, we found that *AfSPL14* could be cleaved by miR156 and miR529 at the same time, consistent with the results of other studies^30^. Furthermore, we found that *AfGAMYB* could be regulated by miR159 and miR319. The expression of two auxin signal-related transcription factors, *AfARF18* and *AfARF12*, was regulated by miR160 and miR167.

### GO and KEGG analysis

**Figure 5:**
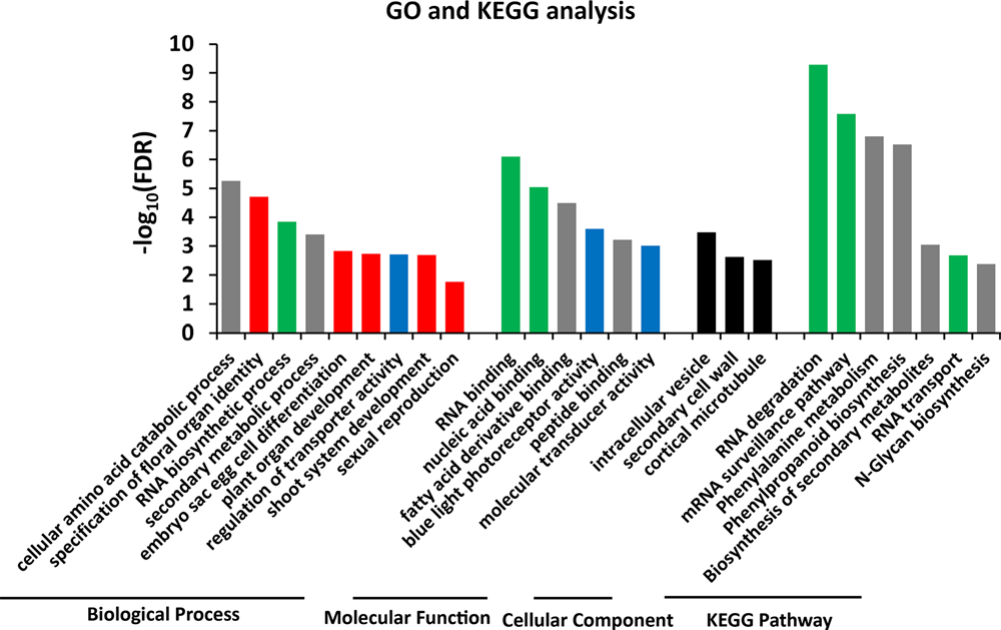
GO and KEGG analysis of target genes. A total of 1,386 target genes from 78 DEMs were used for GO and KEGG analysis. The red columns represent pathways related to developmental processes, the grey columns indicate pathways related to various substance metabolism, the green columns refer to pathways associated with nucleic acid metabolism, the blue columns represent pathways related to molecular activity, and the black columns are mainly related to plant cytoskeleton morphogenesis. All pathways were screened at FDR < 0.05.

To further dissect the function of the identified target genes, Gene Ontology (GO) and KEGG pathway analysis were performed. When the predicted target genes were subjected to GO and KEGG analysis, many genes associated with metabolic substances, transport, nucleic acid-related metabolism (including RNA degradation and DNA polymerase) and responses to biotic stimuli were found to be enriched (Fig. S1). To refine and extend our analysis, a total of 1,386 target genes identified based on 78 corresponding DEMs were screened independently under screening conditions of FDR < 0.001 and |log_2_ (fold change)| > 1 (Table S6). The results showed that many flowering- and floral organ identity-related processes were enriched in GO biological process terms (Fig. 5, red). Nucleic acid (Fig. 5, green) and substance metabolism (Fig. 5, grey) were also found to be obviously enriched and together represented the majority of the terms. Moreover, three molecular activities and some cellular processes were also found to be enriched (Fig. 5, blue and black).

### Integrated expression analysis of miRNAs and their target genes

**Figure 6:**
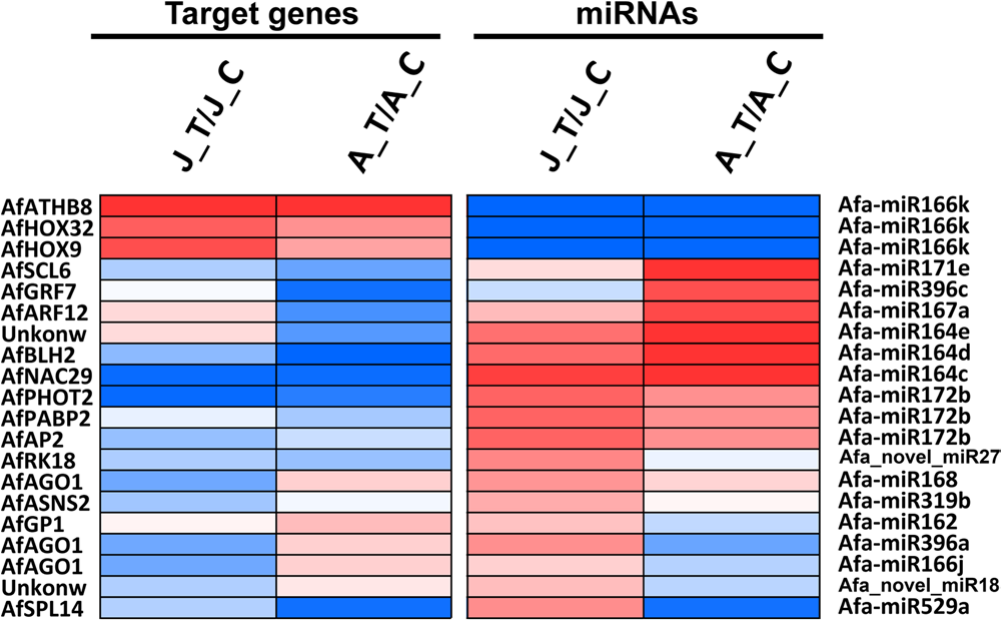
Integrated expression analysis of target genes and miRNAs. All gene expression data were obtained from a previous transcriptome study. RPKM values were used, gene expression changes were calculated by log_2_[ratio (sample1/sample2)], and the heatmap was constructed using Genesis (http://genome.tugraz.at/). The gene names are as follows: *AfGRF7*, *Growth-regulating factor 7*; *AfGP1*, *Vegetative cell wall protein gp1*; *AfAGO1*, *Protein argonaute 1*; *AfSCL6*, *Scarecrow-like protein 6*; *AfARF12*, *Auxin response factor 12*; *AfPABP2*, *Polyadenylate-binding protein 2*; *AfAP2*, *Floral homeotic protein APETALA 2*; *AfNAC29*, *NAC transcription factor 29*; *AfBLH2*, *BEL1-like homeodomain protein 2*; *AfHOX32*, *Homeobox-leucine zipper protein 32*; *AfRK18*, 5*0S ribosomal protein L18*; *AfASNS2*, *Asparagine synthetase 2*; *AfATHB8*, *Homeobox-leucine zipper protein ATHB-8*; *AfHOX9*, *Homeobox-leucine zipper protein 9*; *AfPHOT2*, *Phototropin-2*; *AfSPL14*, *Squamosa promoter-binding-like protein 14*.

To further dissect the mechanism through which miRNAs regulate the ethylene-triggered flowering process in *A. fasciata*, we integrated the results of previous transcriptome research^1^ with our small RNA sequencing data to obtain additional useful information. Because miRNA regulation of gene expression is a post-transcriptional process, genes enriched in sexual reproduction- and RNA metabolism-related GO and KEGG processes were chosen for expression analysis. The results showed that the expression trends of most of the target genes were opposite to those of the corresponding miRNAs (Fig. 6). In addition, we found that many flowering-related genes were regulated by miRNAs during ethylene-triggered flowering; these genes included *AfAP2*, which was regulated by miR172, and *AfSPL14*, which was regulated by miR529. A critical gene for gene transcription and miRNA function, *AfAGO1*, which was predicted as the target of miR168, miR166 and miR319, showed expression trends that were relatively opposite those of the corresponding miRNAs. Other genes, including *AfHOX32*, *AfNAC29*, *AfGRF7* and *AfBLH2*, which are related to phytohormone and stress responses, were also predicted to be regulated by various miRNAs. We thus suspected that various miRNAs and their targets participate in the ethylene-triggered flowering process in *A. fasciata*, a schematic was drew to summarize the current results (Fig. S4).

## Discussion

As in many other species in the family Bromeliaceae, the production of flowers, which are the most valuable ornamental part of *A*. *fasciata*, can be induced by ethylene. As typical endogenous non-coding small RNAs, miRNAs have been reported to be involved in diverse developmental processes and stress responses^31,32^. In previous studies, some miRNAs were found to be independently related to the flowering process and to responses to ethylene^23,33–37^, but no evidence has been presented that miRNA functions in the ethylene-triggered flowering process. In this study, we identified the miRNAs involved in this process in *A*. *fasciata* in the hope that this would clarify the molecular mechanisms that control flowering in plants of the family Bromeliaceae.

In past decades, the molecular bases and pathways related to plant flowering have been identified in many species, especially in the model plants like *Arabidopsis* and rice^38–40^. There are 5 main pathways (vernalization/autonomous-, photoperiod-, gibberellin-, ambient- and aging-pathways) universally existing in plant kingdom controlling plant flowering^41^. For the certain species like Bromeliaceae family, there are specific molecular bases responsible for flowering (e.g. ethylene promotes flowering). MiRNA is a newly identified regulator participating in controlling plant flowering^42^. In early studies of how miRNAs regulate the flowering process, two key miRNAs, miR156 and miR172, were found to play critical roles in the age pathway. The level of expression of miR156 was consistently found to decrease in plants prior to entry into the vegetative-reproductive transition, with concomitant increased expression of miR172^21,23,43,44^. Overexpression of miR156 usually delays flowering of *Arabidopsis*, rice and tobacco^44–46^, whereas overexpression of miR172 promotes flowering in *Arabidopsis*^21,43^. In the present study, the expression of both miR156 and miR172 was detected in juvenile and adult plants, and miR172 was relatively more highly expressed than miR156 (Table S3). After ethylene treatment, miR172 expression was up-regulated in both juvenile and adult plants (Fig. 6), and expression of the target gene, *AfAP2*, was down-regulated. Furthermore, miR172 was found to be highly up-regulated during the juvenile-adult transition (Fig. 3A), consistent with the results of previous studies^34^. *AfAP2* has been reported to be related to the flowering process in *A*. *fasciata*^8^. Thus, we speculate that miR172 may act as a basic regulator of the vegetative-reproductive transition and in the ethylene induction of flowering in *A*. *fasciata*.

The *SPL* TFs regulated by the miR156 family are known to positively regulate the juvenile-adult and adult-flowering transitions in *Arabidopsis* and rice^23,47,48^. In this study, a *SPL* TF from *A*. *fasciata*, *AfSPL14*, was found to be regulated by both miR156 and miR529 (Fig. 4). The secondary structures of miR156 and miR529 were compared, and similar mature sequences and structures were found (Fig. S2). In a previous study, it was found that miR529 was lost during the evolution of *Arabidopsis thaliana* but still exists in many other plants^30^. Our findings suggest that miR156 and miR529 might co-regulate flowering in *A*. *fasciata*.

In addition to miR156 and miR172, many other miRNAs found in our research, including miR159, miR171, etc., have been reported to be related to the flowering process. It was reported earlier that overexpression of miR159 down-regulated the expression of *AtMYB33*, which in turn suppressed the expression of *LFY*, leading to delayed flowering of *Arabidopsis* under short-day conditions^22^. Regulation of *AfGAMYB* by miR159 was also found in this study (Fig. 4), and Afa-miR159b and Afa-miR159e were extremely highly expressed in *A*. *fasciata* (Table S3). miR171 controls the expression of *SCL/LOST MERISTEMS* (*LOM*) genes in *Arabidopsis*^49^. Overexpression of *LOM1* under long-day conditions in *Arabidopsis* results in decreased SOC1 accumulation and late flowering^50^. Our results showed that miR171 was up-regulated by ethylene treatment in adult plants and that its corresponding target, *AfSCL6*, was down-regulated (Fig. 6), implying that miR171 might be involved in ethylene-triggered flowering in *A*. *fasciata*.

On the other hand, as the simplest gaseous phytohormone, ethylene regulates many processes that occur during the plant life cycle, such as responses to abiotic and biotic stress, fruit ripening and senescence^51^. The ethylene-triggered flowering process was first observed in pineapple in the 1930s and was then found to exist widely in other bromeliads. In other plants, the relationship between ethylene signalling and flowering remains uncertain. In *Arabidopsis*, ethylene can enhance the accumulation of DELLA protein, which in turn inhibiting flowering via repressing the expression of *LFY* and *SOC1*^13^. In contrast, research on *OsETR2* and *OsCTR2* in rice revealed a positive role of ethylene in the rice floral transition^15,16^. A miRNA regulating ethylene signalling, miR1917, was recently found to regulate the ethylene response through its target *CTR4* in tomato^35^. In *Arabidopsis*, *EIN3* was reported to accelerate age-dependent leaf senescence by directly repressing the transcription of miR164, which regulates the expression of *NAC*^52^. In our research, miR164 was also found to be un-regulated after ethylene treatment of juvenile and adult plants, and a NAC gene, *AfNAC29*, was down-regulated (Fig. 6). These findings imply that many miRNAs participate in signal transduction in response to ethylene treatment and in the regulation of the flowering process in plants; however, the relationship between miRNA, ethylene signal transduction and flowering will require further study.

## Materials and Methods

### Plant materials, growth conditions, ethephon treatment and RNA preparation

The juvenile (6–8 months) and adult (11–14 months) plants of *A. fasciata* used in this study were grown in a greenhouse located in Danzhou, Hainan Province (China). For ethephon treatment (10:00 a.m.), juvenile and adult plants were treated with 400 μl/L of ethephon (active ingredient 40%) for 24 h; control plants were treated with water at the same time. The ambient temperature was 30–32 °C, and 3 biological replicates were set. Because the leaf is the receptor for ethylene treatment and the shoot apical meristem is the centre for the transition from vegetative growth to reproductive development, mixed samples of core leaves and shoot tips were then harvested after 24 h treatment and immediately frozen in liquid nitrogen for later use. Total RNA was extracted from the mixed samples using the traditional CTAB extraction method^53^.

### Small RNA library construction

To ensure the concentration and integrity of RNA, all total RNA samples were sent to Novogene (Beijing, China) for quality testing using an Agilent 2100 Bioanalyzer system (Agilent Technologies, CA, USA). In brief, small RNAs were isolated from total RNA using PAGE gels; they were then purified and ligated to 3′ and 5′ adapters. Subsequently, cDNAs were generated by reverse transcription, and PCR amplification was performed to obtain sufficient fragments for Illumina sequencing. The PCR products were purified and tested to ensure the quality of the library prior to Illumina sequencing.

### Bioinformatics analysis

To obtain clean reads, the output raw data were first filtered by removing reads containing poly-N, reads with 5′ adapter contaminants, reads lacking 3′ adapters or the insert tag, reads containing poly A or T or G or C and reads of length <18 nt. All clean reads were first aligned to the GenBank (ftp://ftp.ncbi.nlm.nih.gov/genbank/) and Rfam 11.0 (http://rfam.janelia.org/) databases, and the reads annotated as rRNA, tRNA, scRNA, snRNA and snoRNA were removed. The remaining clean reads were aligned to the miRBase 21.0 (http://www.mirbase.org/ftp.shtml) to obtain the known miRNAs. Due to the lack of an available genome sequence, transcriptomic sequencing data of *A. fasciata*^1^ were used for novel miRNA prediction using PmiRDiscVali^54^. Novel miRNAs then were identified and annotated according to the reported standards^55^. The normalized expression level of each miRNA was calculated according to the following formula: transcripts per million (TPM) = mapped read count/total reads * 1,000,000. The fold change in expression of miRNAs between samples was calculated using log_2_(TPM of sample 1/TPM of sample 2). DEGseq R package was used for differential expression analysis^56^. The heatmap was generated using Genesis software (http://genome.tugraz.at/) using the fold change values. To further analyse the functions of the miRNAs, their target genes were predicted by psRobot (http://omicslab.genetics.ac.cn/psRobot/) and Target Finder (http://www.mirtoolsgallery.org/miRToolsGallery/) using transcriptomic sequencing data as reference sequences^1^.

### GO and KEGG analysis

To predict the functional category distribution frequency of miRNA target genes, Gene Ontology (GO) term analysis (www.geneontology.org) was applied using Blast2GO software. To explore the differences in pathways, KEGG analysis was also performed with KOBAS 3.0 (http://kobas.cbi.pku.edu.cn/). All GO categories and KEGG pathways were screened at FDR < 0.05.

### Reverse transcription and quantitative reverse transcription PCR (qRT-PCR) analysis

To validate changes in the expression of miRNAs, the stem-loop RT-PCR method was used to quantify the expression levels of miRNAs^57^. Briefly, 4 μg of total RNA was first mixed with 0.05 μM stem-loop primers (synthesized by Genscript Bioscience, Nanjing, China), 2.5 μM oligo dT primer and an appropriate volume of RNAase-free water, incubated at 65 °C for 5 min, and then transferred to ice for 2 min. The mixture was then added to 4 μl of 5× first-strand buffer, 1 μl of 0.4 μM dNTP, 1 μl of dithiothreitol (DTT; 100 mM), 1 μl of RNaseOUT (40 units/μl) and 1 μl of SuperScript III RT (200 units/μl, Invitrogen, Carlsbad, CA)) in a 20-μl reaction. The reverse-transcription program consisted of the following steps: 16 °C for 30 min; 60 cycles of 30 °C for 30 sec, 42 °C for 30 sec and 50 °C for 1 sec; and a final incubation at 85 °C for 5 min to inactivate the reverse transcriptase.

qRT-PCR was performed with 10 μl of 50× diluted cDNA products, 10 μl of TransStart Tip Green qPCR SuperMix (Transgen, Beijing, China) and 0.25 μM forward and reverse primers using a Therma PikoReal 96™ Real-Time PCR System (Thermo Fisher Scientific, New York, USA). Thermal cycling was conducted for 30 sec at 95 °C followed by 40 cycles of 5 s at 95 °C and 35 s at 60 °C. For each miRNA, three biological replicates and three technical replicates were included. The *α-actin* gene from *A. fasciata* was used as an internal standard. Relative miRNA levels were calculated using the 2^−△△CT^ method. The stem-loop and qRT-PCR primers used in this study are listed in Table S9.

### miRNA northern blotting

To validate the expression of miRNAs, northern blotting was performed according to the methods described in previous studies^58^. In briefly, 20 μg of total RNA from each sample was separated on a 15% polyacrylamide gel and transferred to an Immobilon-Ny+ membrane (Merck Millipore, http://www.merckmillipore.com). Subsequently, the membrane was hybridized with probes labelled with c32P-ATP at 37 °C overnight in Hybridization Solution (TOYOBO, http://www.toyobo-global.com). Finally, the membrane was washed several times with low- (19 SSC, 0.5% SDS) and high-stringency (0.29 SSC, 0.2% SDS) buffer at 37 °C and exposed using a phosphorimager. The probe sequences are listed in Table S9.

### RNA ligase-mediated rapid amplification of cDNA ends (RLM-RACE)

RLM-RACE was performed using a GeneRacer kit (Invitrogen, USA) to validate the accuracy of the predicted target genes of the miRNAs. Briefly, total RNA (5 μg) from core leaves and shoot tips was first ligated to RNA adapters without the use of calf intestine alkaline phosphatase. The cDNA was then transcribed using GeneRacer Oligo dT primers. PCR was performed with 5′ adapter primers (sequence provided by the manufacturer) and 3′ gene-specific primers (designed by Primer 5.0 and synthesized by Genscript, Nanjing, China). The RACE products were ligated to a T-easy vector and sequenced by Tianyi Huiyuan (Guangzhou, China).

## Supplementary information

Supplementary information

Supplementary information 2

## Data Availability

Small RNA sequence data from core leaves and shoot tips sampled from plants that received mock control and ethephon treatment have been submitted to the NCBI Sequence Read Archive (BioProject: PRJNA435887).
