# Supplementary material for: Small RNA sequencing revealed various microRNAs involved in ethylene-triggered flowering process in Aechmea fasciata
Source: Sci Rep. 2020 Apr 30;10:7348. doi: 10.1038/s41598-020-63597-1 (PMC7193560; doi:10.1038/s41598-020-63597-1)
Supplement: Supplementary file 1 — Supplementary information [file 41598_2020_63597_MOESM1_ESM.pdf]

Small RNA sequencing revealed various microRNAs involved in ethylene-triggered flowering process in *Aechmea fasciata*

Authors: Yuanhao Ding[1, 2, 3 $], Jiabin Wang[1, 2 $], Ming Lei[1, 2], Zhiying Li[1, 2], Yonglin Jing[1, 2], Haiyan Hu[3], Sitao Zhu[4], Li Xu[1, 2, *]

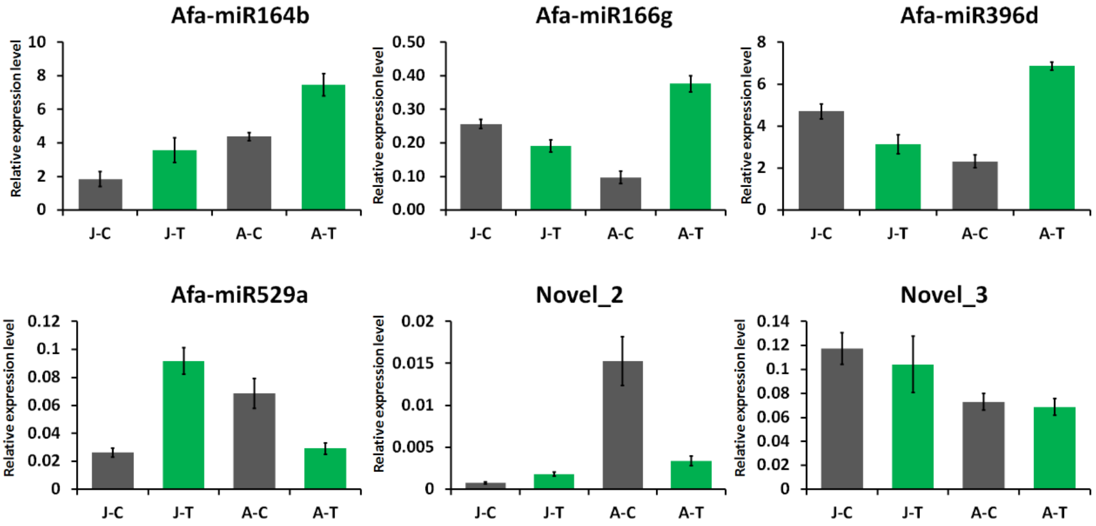

Figure S1: Validation of the relative expression of miRNAs by qRT-PCR.

The relative expression levels of 6 differentially expressed miRNAs are shown. An α-actin gene from A. fasciata was used as an internal control.

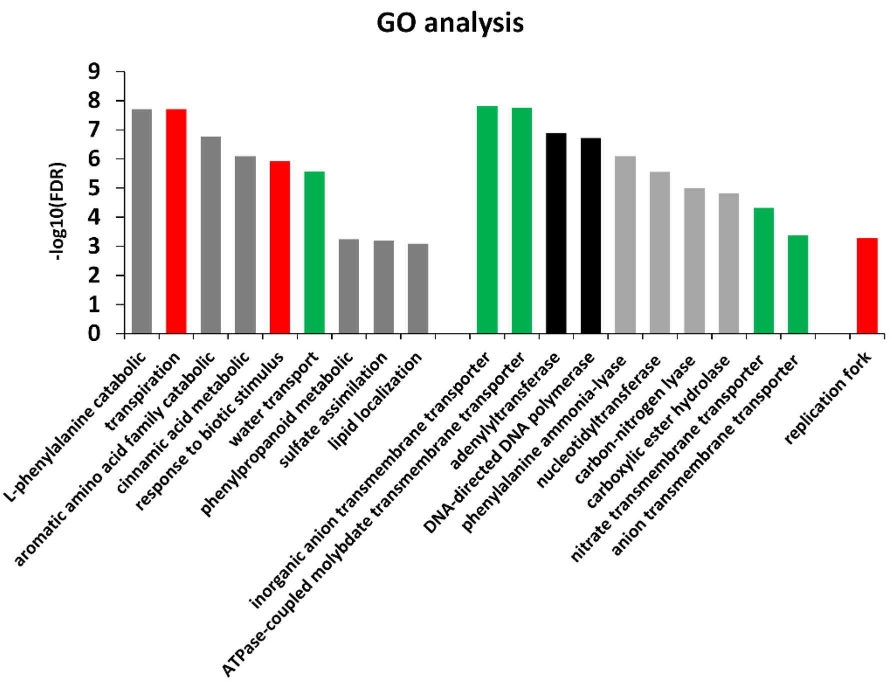

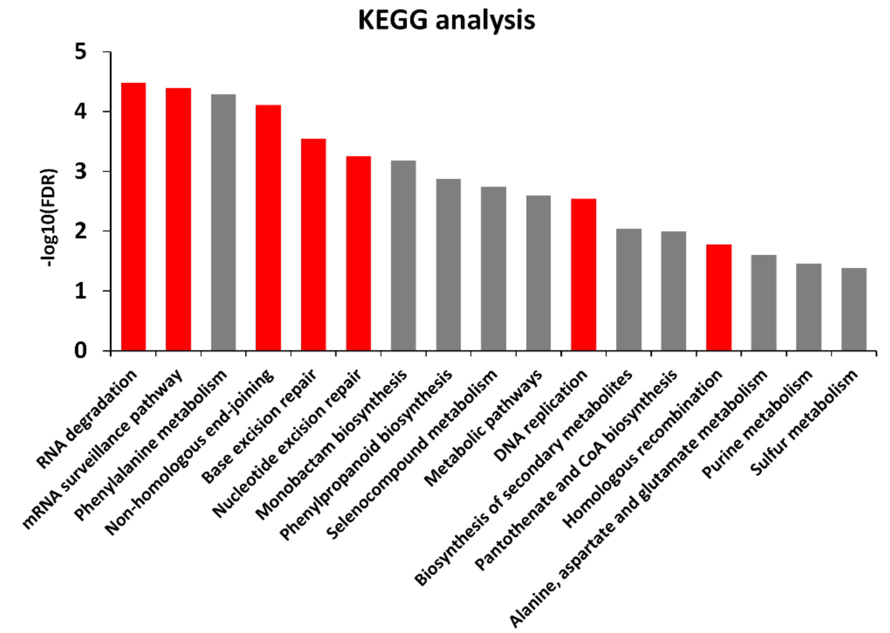

Figure S2: GO and KEGG analysis of predicted targets.

A total of 1719 target genes predicted from 115 miRNAs were used for GO and KEGG analysis. All pathways were screened at FDR < 0.05.

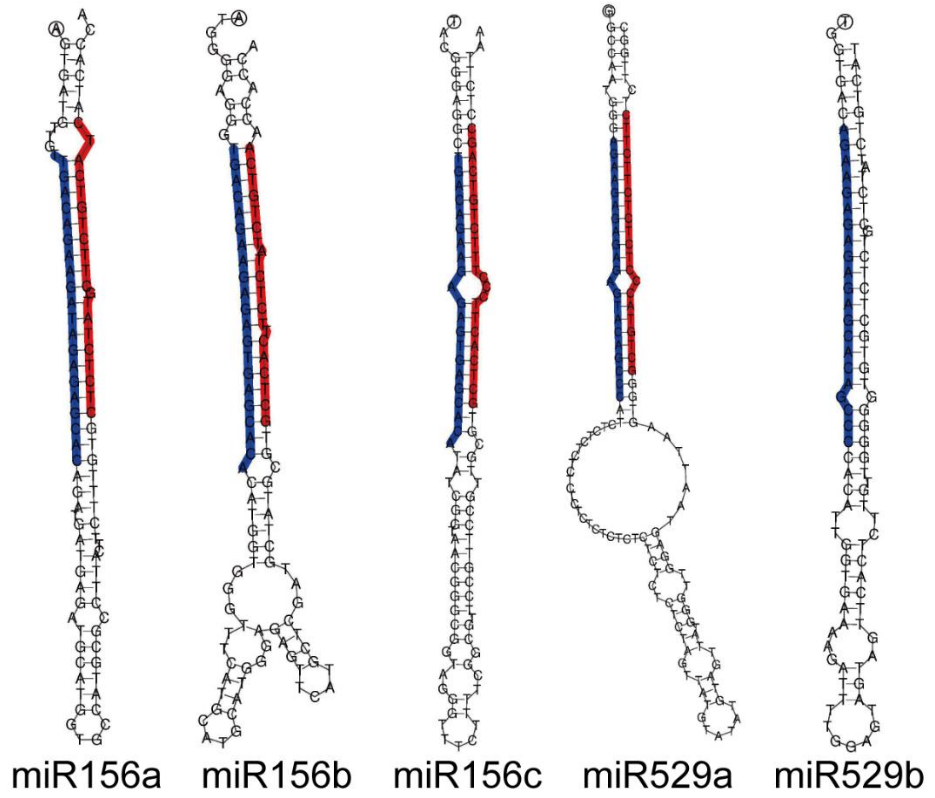

Figure S3: Secondary structures of the miR156 and miR529 families.

The secondary structures of 3 members of the miR156 family and 2 members of the miR529 family are shown.

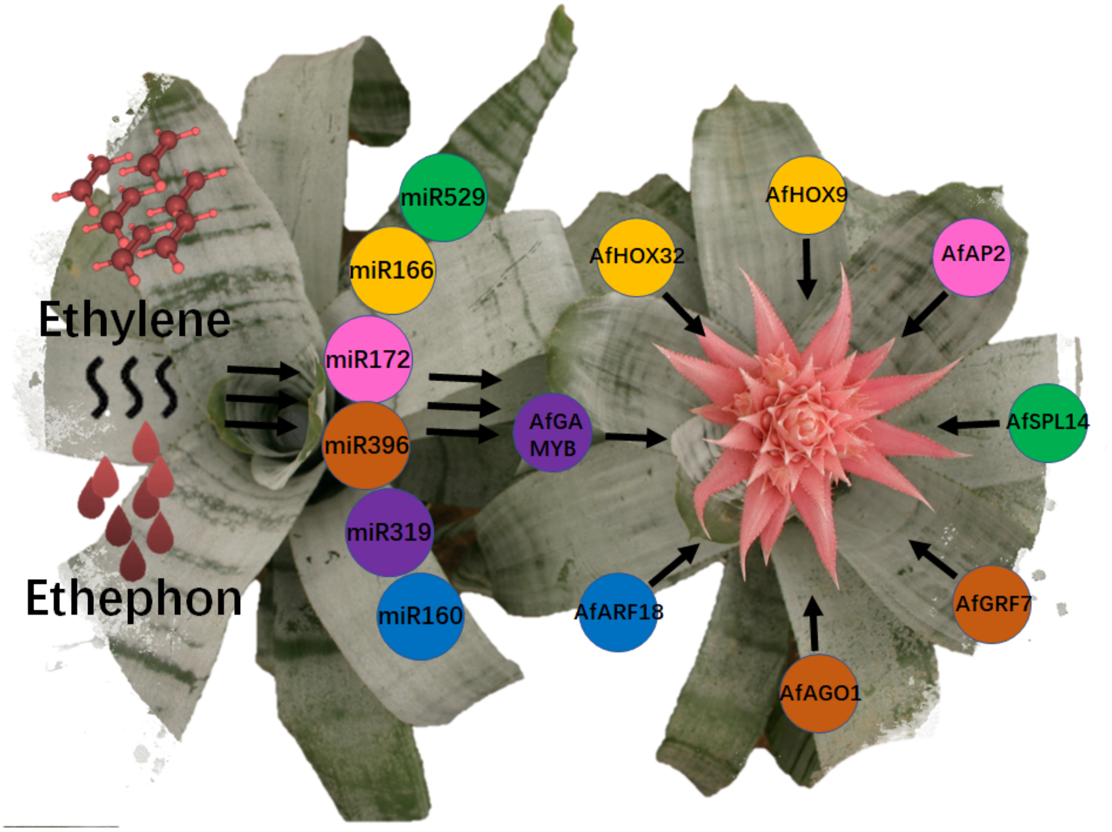

Figure S4: A schematic illustration showed miRNAs and target genes involved in ethylene-triggered flowering of *A. fasciata*.
